# Extracellular vesicles shed from gastric cancer mediate protumor macrophage differentiation

**DOI:** 10.1186/s12885-021-07816-6

**Published:** 2021-01-28

**Authors:** Atene Ito, Shunsuke Kagawa, Shuichi Sakamoto, Kazuya Kuwada, Hiroki Kajioka, Masashi Yoshimoto, Satoru Kikuchi, Shinji Kuroda, Ryuichi Yoshida, Hiroshi Tazawa, Toshiyoshi Fujiwara

**Affiliations:** 1 Department of Gastroenterological Surgery, Okayama University Graduate School of Medicine, Dentistry and Pharmaceutical Sciences, 2-5-1 Shikata-cho, Kita-ku, Okayama, 700-8558 Japan; 2 Minimally Invasive Therapy Center, Okayama University Hospital, Okayama, Japan; 3 Center for Innovative Clinical Medicine, Okayama University Hospital, Okayama, Japan

**Keywords:** Extracellular vesicles, Gastric cancer, Tumor-associated macrophages, Tumor microenvironment

## Abstract

**Background:**

Peritoneal dissemination often develops in gastric cancer. Tumor-associated macrophages (TAMs) are present in the peritoneal cavity of gastric cancer patients with peritoneal dissemination, facilitating tumor progression. However, the mechanism by which macrophages differentiate into tumor-associated macrophages in the peritoneal cavity is not well understood. In this study, the interplay between gastric cancer-derived extracellular vesicles (EVs) and macrophages was investigated.

**Methods:**

The association between macrophages and EVs in peritoneal ascitic fluid of gastric cancer patients, or from gastric cancer cell lines was examined, and their roles in differentiation of macrophages and potentiation of the malignancy of gastric cancer were further explored.

**Results:**

Immunofluorescent assays of the ascitic fluid showed that M2 macrophages were predominant along with the cancer cells in the peritoneal cavity. EVs purified from gastric cancer cells, as well as malignant ascitic fluid, differentiated peripheral blood mononuclear cell-derived macrophages into the M2-like phenotype, which was demonstrated by their morphology and expression of CD163/206. The macrophages differentiated by gastric cancer-derived EVs promoted the migration ability of gastric cancer cells, and the EVs carried STAT3 protein.

**Conclusion:**

EVs derived from gastric cancer play a role by affecting macrophage phenotypes, suggesting that this may be a part of the underlying mechanism that forms the intraperitoneal cancer microenvironment.

**Supplementary Information:**

The online version contains supplementary material available at 10.1186/s12885-021-07816-6.

## Background

Gastric cancer (GC) is one of the most common cancers and the third leading cause of cancer-related death in the world [1]. Peritoneal dissemination is one of the most common metastatic patterns of GC [2], and it results in a poor prognosis, but its underlying mechanism remains unclear. In the tumor microenvironment (TME), co-stimulation between normal cells and cancer cells is reported to be important in promoting malignancy [3, 4]. Macrophages physiologically play roles in dead cell destruction, vessel formation, and inflammation induction [5], and they are also one of the key players even in the TME, where they are called tumor-associated macrophages (TAMs). Macrophages are classified into dichotomous phenotypes: the classical M1 type and the alternative M2 type macrophages. M1 type macrophages have a role in anti-tumor immunity and the inflammatory response. On the other hand, M2 type macrophages are involved in the anti-inflammatory response, wound healing, and pro-tumorigenic properties. TAMs are generally considered to more closely resemble the M2-type and thereby modulate the pro-tumor microenvironment [6]. Thus, TAMs in tumor tissues have been shown to be prognostic biomarkers for various cancers [7]. In addition, targeting TAMs has been investigated in clinical studies [7–9].

Extracellular vesicles (EVs) play an essential role in intercellular communication between tumor and surrounding stromal cells or even between tumor and distant cells. In general, EVs, including exosomes and microvesicles, are small membrane vesicles that contain various carriers, such as microRNAs (miRNAs), messenger RNAs, and proteins [10–13]; they can be released from various types of cells and function in communication between cells through transferring their contents [14, 15]. Accumulating evidence has shown that EVs secreted from cancer cells affect surrounding cells and even cells at distal sites, thereby enabling the development of a TME that promotes tumor growth [16–19]. For instance, tumor exosomal integrins could determine organotrophic metastasis [20], and EVs secreted from GC also deliver EGFR, which could induce liver metastasis [21]. Furthermore, Wu et al. reported that EVs from GC activate macrophages to promote cancer progression [22]. These reports suggested a critical pro-tumor role of cancer-derived EVs in the TME [23, 24].

Recent studies by us and other investigators have shown that macrophages in the peritoneal cavity, especially M2-type macrophages, could contribute to progression of GC with peritoneal dissemination [25, 26]. However, the mechanisms of how macrophages change their phenotype in the microenvironment remain unclear. We hypothesized that EVs are secreted from GC cells in the peritoneal cavity, and they might affect the phenotype of macrophages to promote dissemination.

In this study, the interplay between GC-derived EVs and macrophages was investigated, and the background mechanism involved in the formation of intraperitoneal cancer microenvironment was evaluated.

## Methods

### Cell culture

The GC cell line, GCIY, was purchased from RIKEN (Saitama, Japan) and cultured in MEME (Sigma-Aldrich, St. Louis, MO, USA) supplemented with 15% fetal bovine serum (FBS) and 1% penicillin/streptomycin (Sigma-Aldrich), and other GC lines, MKN7 and MKN45, were purchased from the Japanese Collection of Research Bioresources cell bank and cultured in RPMI1640 (Sigma-Aldrich) supplemented with 10% FBS and 1% penicillin/streptomycin. The human mesothelial cell line (MeT-5A) was purchased from the American Type Culture Collection (ATCC, Manassas, VA, USA) and cultured in McCoy’s 5A medium (Thermo Fisher Scientific, Tokyo, Japan) supplemented with 10% FBS and 1% penicillin/streptomycin. All cells were maintained at 37 °C in a humidified atmosphere with 5% CO_2_. We purchased the cell lines used in the experiments directly from the authorized distributors and confirmed that all cell lines were uncontaminated in the public databases. Additional information on cell lines is provided in the Supplementary Table.

### Preparation of EVs

EVs were isolated by ultracentrifugation according to the methods previously reported [27]. Briefly, cells were washed with PBS twice and cultured in the medium with exosome-depleted fetal bovine serum (after overnight centrifugation at 100,000 g) for 48 h. The conditioned medium (CM) was collected and centrifuged at 2000 g for 10 min, and the supernatant was filtered through a 0.22-um filter (Millipore®, Merck, Tokyo, Japan). The CM was then ultracentrifuged at 100,000 g for 70 min, and the pellet was finally washed by PBS and centrifuged at 100,000 g for 70 min.

To prepare EVs from clinical samples, peritoneal washings or ascitic fluid was collected from patients who gave their informed consent to participate in the study [28, 29]. Peritoneal washings or ascitic fluid were obtained during operation for the purpose of cytology. Ascites, if present, is aspirated; if not, 100 ~ 200 mL of normal saline is instilled into the Douglas pouch, gently stirred, and then aspirated as peritoneal washings. Half of it was subjected to pathological cytology and the other half was stored at − 80 °C for the study. Those used in this study were selected retrospectively in consideration of stock status and clinical stages. The clinicopathological data were obtained from the medical records, and the cancers were staged in accordance with the Japanese Classification of Gastric Carcinoma: 3rd English edition [30]. After thawing the frozen samples, first, peritoneal washings or ascitic fluid was centrifuged at 300 g for 5 min. The supernatant was again centrifuged at 2000 g for 10 min to remove dead cells and debris. The supernatant was centrifuged at 10,000 g for 30 min to remove large EVs. Finally, the supernatant was ultracentrifuged at 100,000 g for 70 min, and the pellet was washed by PBS and centrifuged at 100,000 g for 70 min. After the EVs were resuspended in PBS, the putative protein amount was measured by Micro BCA Protein Assay (Thermo Fisher Scientific) according to the manufacturer’s protocol, and vesicle size was measured by Zetasizer (Malvern Panalytical Ltd., Malvern, United Kingdom).

### Transmission electron microscopy (TEM)

Briefly, 10 μl of a suspension of EVs were placed on a carbon coated copper grid for 15 min, and excess suspension liquid was removed. The samples were negatively stained with 2% uranyl acetate solution for 2 min. After air drying, the samples were visualized using an H-7650 transmission electron microscope (Hitachi High-technologies, Tokyo, Japan) in the Central Research Laboratory, Okayama University Medical School.

### Monocyte and macrophage preparation from peripheral blood mononuclear cells (PBMCs)

PBMCs were collected from whole blood of healthy donors. PBMCs were obtained from buffy coats by Ficoll-Paque (GE Healthcare Biosciences, Piscataway, NJ; catalog 17–1440-02) density gradient centrifugation at 400 g at 20 °C for 40 min to separate blood constituent parts. After purified cells were washed with PBS, CD14+ monocytes were isolated using the Pan Monocyte Isolation kit (Miltenyl Biotec, San Diego, CA; catalog 130–096-537) followed by separation using magnetic LS columns (Miltenyl Biotec; catalog 130–042-401), according to the manufacturer’s protocol.

Macrophages were differentiated from these monocytes according to the previous reports [31, 32]. The monocytes were cultured in RPMI1640 with either GM-CSF at 20 ng/ml (named M0-GM) or M-CSF at 20 ng/ml (named M0-M) for 5 days. M0-GM macrophages were then stimulated by LPS (20 ng / ml) and IFN-γ (20 ng / ml) for 4 days to polarize into CD80+ / CD86+ (M1) macrophages. On the other hand, M0-M macrophages were stimulated by IL-4 (20 ng / ml) and IL-13 (20 ng / ml) for 4 days to polarize into CD163+ or CD206+ (M2) macrophages (Fig. S3A-C). M0-GM or M0-M macrophages were stimulated by EVs (20 μg / ml) for 4 days to assess the effects of EVs.

### EVs uptake

CD14+ cells were purified from PBMCs. Collected EVs from each cell line were labelled with the PKH26 Red Fluorescent Cell Linker Mini Kit (Sigma-Aldrich) for visualization according to the manufacturer’s protocol. EVs were incubated with 2 μM of PKH26 for 5 min and washed twice. To remove excess dye, Exosome Spin Columns (MW3000) (Thermo Fisher Scientific) were used. PKH26-labeled EVs were used to confirm EV uptake in monocytes or macrophages. Cells were incubated with PKH26-labeled EVs purified from GCIY culture medium (GCIY-EVs) and analyzed by fluorescence microscopy and flow cytometry.

### Immunofluorescence staining

**Fig. 1:**
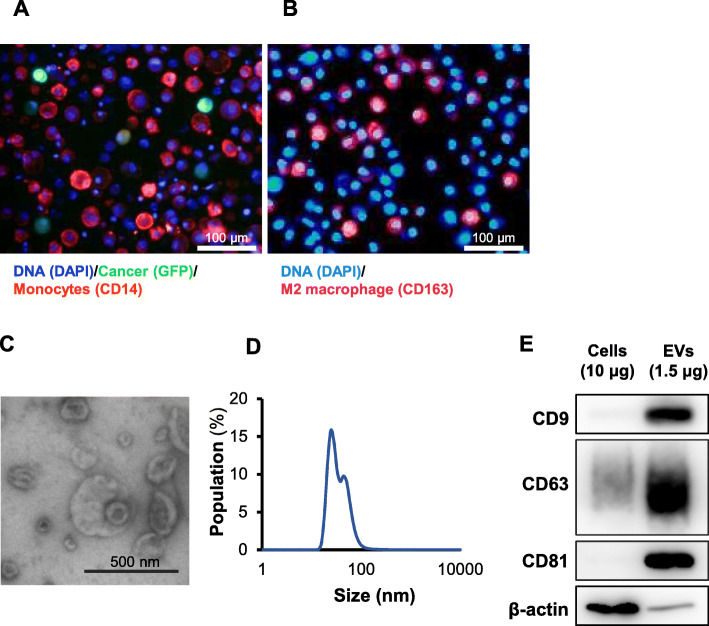
CD163-positive macrophages predominate in both peritoneal washings from a cytology-positive GC patient and malignant ascitic fluid. EVs were contained in malignant ascitic fluid. **a** and **b** are immunofluorescent images of the same malignant ascitic fluid. **a** Cancer cells and monocytes were imaged by the cancer-imaging virus OBP-401 (GFP) and anti-CD14 antibody (PE), respectively. Macrophages were present in malignant ascitic fluid. **b** M2 macrophages were imaged by anti-CD163 antibody (PE). Scale bar: 100 μm. **c** Transmission electron microscope image of EVs from MA. Scale bar: 500 nm. **d** Vesicle size measured by dynamic light scattering analyses. **e** Representative Western blot of cells and EVs in MA to confirm the presence of EVs by exosome markers (CD9, CD63, CD81). Full-length blots / gels are presented in Fig. S5

Malignant ascitic fluid or peritoneal washings were centrifuged to collect cells, and the cells were resuspended in RPMI-1640 supplemented with 10% FBS. The number of viable cells was counted, and the cancer cells were labeled with GFP by infection with the cancer-imaging virus OBP-401 at 1 multiplicity of infection for 24 h at 37 °C. Cancer cells were detected as GFP-positive cells [33–35]. Macrophages were stained with PE or Alexa Fluor 647-conjugated anti-human CD14 antibody (BioLegend, San Diego, CA, USA) as a pan-macrophage marker and PE-conjugated anti-human CD163 antibody (BioLegend) as a marker of M2 macrophages, respectively. The nucleus was stained with 4′, 6-diamidino-2-phenylindole (DAPI). Stained cells were observed under an inverted fluorescence microscope (IX71; Olympus, Tokyo, Japan) equipped with the DP70 camera. The images were captured using the DP controller software (Olympus). The imaging using OBP-401 was applied only for Fig. 1 a and b.

### Mouse peritoneal dissemination model

Female BALB/c (nu / nu) mice were purchased from CLEA (Tokyo, Japan). Five million of each of the cancer cells including GCIY, MKN7, and MKN45 were inoculated into the peritoneal cavity (*n* = 5 / group, *n* = 15 in total) when the mice were 6 weeks of age. Tumor burdens were evaluated by an IVIS Spectrum system (Caliper Life Sciences, Waltham, MA, USA) every week. After the experiment, the mice were euthanized by carbon dioxide inhalation followed by cervical dislocation.

### Flow cytometry

Collected cells were washed twice and suspended in PBS with 2% FBS before staining. Cells were evaluated by surface markers using directly-labeled primary monoclonal antibody (mAb) CD80-PE / Cy7, CD86-APC, CD163-PE, CD206-FITC, and the appropriate isotype-controls for each antibody (BioLegend). After cells were stained for 30 min, they were washed twice and suspended in PBS. These samples were subjected to BD FACS Lyric (BD Biosciences), and the data were analyzed using FlowJo software (Becton, Dickinson and Company, Ashland, OR, USA). Viability was analyzed assay by the Annexin V-FITC Early Apoptosis Detection Kit (Cell signaling Technology) according to the manufacturer’s protocol.

### Western blot

Equivalent amounts of protein from whole-cell lysates or EVs were loaded into each lane of SDS-polyacrylamide gel and electrophoretically transferred to polyvinylidene difluoride membranes (Hybond-P; GE Health Care, Buckinghamshire, UK). Membranes were incubated in Blocking One or Blocking One-P (Nacalai Tesque, Inc., Kyoto, Japan) for 30 min before being incubated in the following primary antibodies overnight at 4 °C: anti-CD9 (Chemicon International Inc., Temecula, CA, USA), anti-CD63 (BD Biosciences), anti-CD81 (BioLegend), anti-signal transducer and activator of transcription 3 (STAT3; Cell Signaling Technology), anti-phosphorylated STAT3 (p-STAT3; Cell Signaling Technology), anti-Akt (Akt; Cell Signaling Technology), anti-phosphorylated Akt (p-Akt; Cell Signaling Technology), and anti-β-actin antibody (Sigma-Aldrich). The membranes were subsequently incubated with secondary antibodies for 60 min at room temperature. Peroxidase activity of secondary antibodies was detected using ECL prime Western Blotting Detection Reagent (GE Healthcare UK Ltd.) and visualized using an Amersham Imager 600 (GE Healthcare UK Ltd.) according to the manufacturer’s protocol.

### Migration assay

The migration assay was performed using Transwell 24-well plates with 8-μm-pore polyethylene terephthalate (PET) track-etched membranes (CORNING). GC cells were indirectly co-cultured with macrophages stimulated by various EVs (ratio 1: 2) using Transwell 6-well plates with 4-μm-pore PET track-etched membranes (CORNING) for 48 h. Then, 1 × 10^5^ cells of MKN7 were seeded in the upper chamber with 500 μl of RPMI1640 containing 0.1% FBS. The lower chamber was filled with RPMI1640 containing 10% FBS. After incubation for 24 h at 37 °C, migrating cells were fixed with 4% paraformaldehyde and stained with 0.5% crystal violet. The number of cells on the lower surface of the upper chamber membrane in 5 random fields was counted using a bright field light microscope.

### ELISA for human IL-6

The IL-6 levels secreted by macrophages were determined using an enzyme-linked immunosorbent assay (ELISA) kit (Human IL-6, catalog # D6050, R&D Systems, Minneapolis, MN, USA) according to the manufacturer’s instructions.

### Statistical analysis

Unless otherwise stated, results are expressed as means ± S.D. Student’s *t*-test was used to compare continuous data, and a *P* value less than 0.05 was considered significant. All statistical analyses were conducted with EZR (Saitama Medical Center, Jichi Medical University), which is a graphical user interface for R (The R Foundation for Statistical Computing, version 2.13.0).

## Results

### CD163 + macrophages and extracellular vesicles were detected in cytology-positive peritoneal washings or malignant ascitic fluid from gastric cancer patients

Since it has been reported that intraperitoneal macrophages are preferentially differentiated into M2 macrophages in GC patients, cellular components in peritoneal washings in a case of a peritoneal cytology-positive GC patient were investigated. It was confirmed that numerous CD14+ cells existed in the peritoneal cavity along with GFP-positive cancer cells (Fig. 1a). When they were stained with anti-CD163 antibody, abundant CD163+, M2-type, macrophages were present (Fig. 1b). Such results let us to investigate how these intraperitoneal tumor-associated macrophages were skewed to M2 types.

EVs were reported to function in the intercellular communications, so we then attempted to extract EVs from malignant ascites and peritoneal washings to verify whether EVs were present in the peritoneal cavity. EVs were purified from malignant ascitic fluid derived from GC patients with cancer dissemination (Fig. 1d-f) or peritoneal washings collected during surgery for a GC patient (Fig. S1 A, B), indicating that a certain amount of EVs existed in the peritoneal cavity in GC patients. It was presumed that some of these EVs were derived from GC cells, which prompted us to further investigate the possible interaction of EVs derived from GC cells and intraperitoneal macrophages.

### EVs from GC cell lines sustained the viability of monocytes and induced PD-L1 expression on these surviving monocytes

**Fig. 2:**
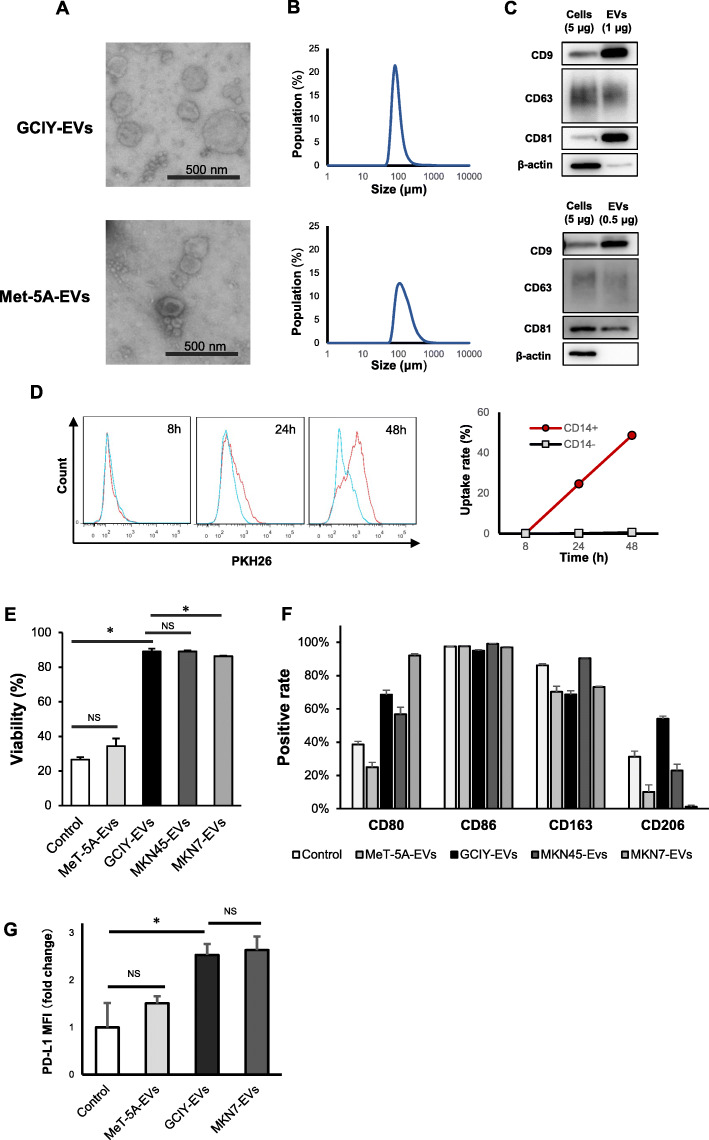
Extracellular vesicles derived from GCIY (upper) and MeT-5A (lower). **a** Transmission electron microscope image of EVs. Scale bar: 500 nm. **b** Vesicle size measured by dynamic light scattering analyses. **c** Representative Western blot of original cell lysates and EVs to confirm the presence of EVs by exosome markers (CD9, CD63, CD81). Full-length blots / gels are presented in Fig. S6 and S7. **d** The uptake of GCIY-EVs into CD14-positive cells (monocytes) was analyzed by flow cytometry. **e**-**g** Flow cytometry analysis for monocytes co-cultured with EVs for 48 h. *N* = 3, in each samples. **e** The viability of monocytes treated with each EVs. **f** The positive rates of the markers on each monocyte. **g** PD-L1 expression on each monocyte. (*: *p* < 0.05, NS: not significant, Student’s t-test)

Although macrophages differentiate from monocytes, when and how macrophages were further differentiated to M1 or M2 types in the peritoneal environment were unknown. Therefore, the effect of EVs from GC cell lines on macrophages was examined in vitro. To compare the functionality of EVs between EVs from normal cells and EVs from cancer cells, EVs were purified from a normal mesothelial cell line (MeT-5A) and 3 GC cell lines, GCIY, MKN7, and MKN45 (Fig. 2a-c, Fig. S2A-C). These 3 GC cell lines were chosen from among the available lines, because we have previously confirmed that these cells displayed different aggressiveness in terms of tumorigenesis. GCIY and MKN45 cells develop peritoneal dissemination after peritoneal injections, but MKN7 cells do not (Fig. S2D). In addition, GCIY cells grow more aggressively than MKN45 cells (Fig. S2D).

Whether EVs affect CD14+ monocytes, precursors of macrophages, was investigated first. More than 50% of monocytes could take up EVs purified from GCIY culture medium (GCIY-EVs) in 48 h (Fig. 2d and Fig. S2E), but, on the other hand, CD14-negative cells (mainly lymphocytes) could not take up GCIY-EVs (Fig. S2F). The viability assay and surface marker analysis for monocytes treated with EVs demonstrated that monocytes could sustain high viability with GCIY-EVs, MNK45-EVs or MKN7-EVs, while about 80% of monocytes died without the GC-derived EVs (Fig. 2e). Such surviving monocytes highly expressed CD86, as well as CD163 (Fig. 2f), with or without GC-derived EVs. The expression of CD206 was found to be increased by GCIY-EVs, but not by MET-5A-EVs, MKN-45-EVs or MKN7-EVs (Fig. 2f). The expression of PD-L1 on the surface of monocytes was found to be increased by GCIY-EVs and MKN7-EVs (Fig. 2g).

GC cell-derived EVs (GC-EVs) affected monocytes to increase the sustainability and expression of CD206 and PD-L1, suggesting that GC-EVs might be associated with skewing macrophage phenotype.

### Macrophages derived from PBMCs were skewed to an M2-like phenotype by GC-EVs, and these macrophages promoted the malignancy of cancer cells by secreting IL-6

**Fig. 3:**
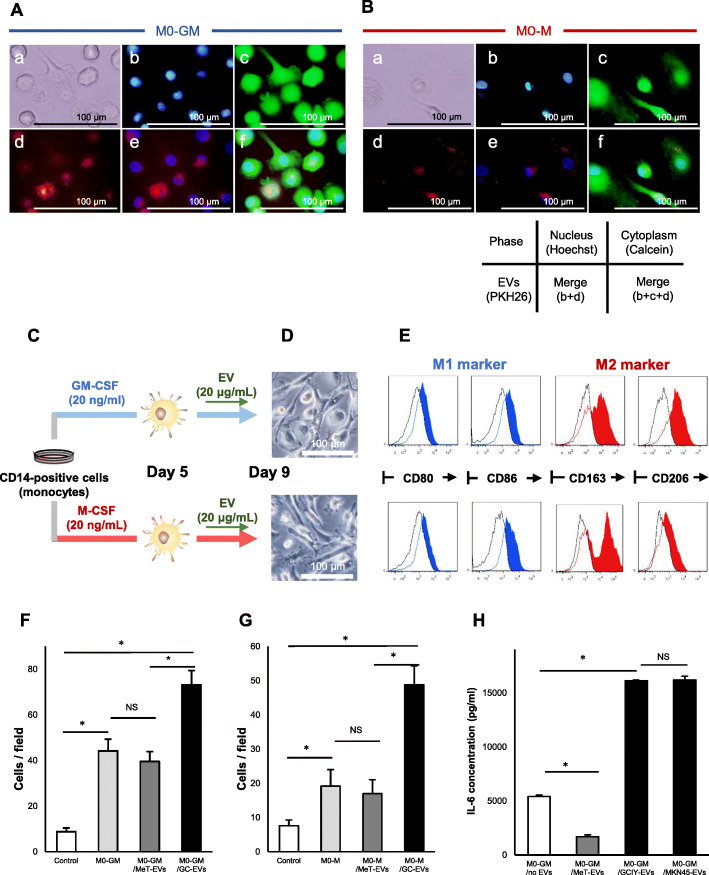
PBMCs-derived macrophages that were polarized into M2 type by treatment with GCIY-EVs promoted malignancy of cancer cells via IL-6 secretion. **a**, **b** The uptake of EVs by day 5-macrophages is visualized. M0-GM Macrophages (**a**) and M0-M macrophages (**b**) were stained by Calcein for the cytoplasm, and Hoechst for the nucleus. EVs were stained by PKH26 before co-culture. Magnification, × 200. bar: 100 μm. **c** Protocol for differentiation of monocytes. **d** Morphology of M0-GM and M0-M macrophages. **e** Surface markers of macrophages treated with GCIY-EVs. Upper figures are macrophages treated from M0-GM and lower are from M0-M. CD80 and CD86 were adopted as M1 markers, and CD163 and CD206 as M2 markers. Scale Bar: 100 μm. **f** Migration assay of MKN7 cells co-cultured with M0-GM macrophages treated by EVs. Control: Cancer cells only. M0-GM: Cancer cells co-cultured with untreated macrophages. M0-GM/MET-EVs: Cancer cells co-cultured with macrophages treated with MET-5A cells-derived EVs. M0-GM/GC-EVs: Cancer cells co-cultured with macrophages treated with GCIY cell-derived EVs. **g** Migration assay of MKN7 cells co-cultured with M0-M macrophages treated by EVs. **h** Concentration of IL-6 in the supernatant of macrophages treated with each EV was analyzed by ELISA assay. (*: *p* < 0.05, NS: not significant, Student’s t-test)

The effect of GC-EVs on cells at different stages in the process of monocyte-macrophage differentiation was further examined. Before the analysis, it was confirmed that M1 or M2 macrophages could differentiate from PBMC-derived CD14+ monocytes by GM-CSF or M-CSF stimulation, in terms of morphology and surface markers (Fig. S3A-C). Based on that, monocytes on day 5, just after GM-CSF or M-CSF treatment, were regarded as immature macrophages and named M0-GM or M0-M, respectively. The uptake of GC-EVs into day 5 macrophages (M0-GM or M0-M) was then checked using PKH26-labeled EVs. The EVs were taken up by both macrophages after incubation for only 3 h (Fig. 3a and b). Next, M0-GM and M0-M macrophages were treated with GCIY-EVs for 4 days, and the surface markers of these macrophages were observed. After the treatment with GCIY-EVs, the macrophages expressed CD80 and CD86 weakly and CD163 and CD206 strongly, regardless of preconditioning with GM-CSF or M-CSF (Fig. 3c-e).

From the above results, it was found that GC-EVs promoted the differentiation of macrophages into M2 type, and we next examined whether the properties of macrophages could be altered by the malignant characters of gastric cancer cells from which EVs were derived. Although GCIY and MKN45 cells develop peritoneal dissemination after peritoneal injections, GCIY cells grow more aggressively than MKN45 cells. In contrast, MKN7 cells could not develop peritoneal dissemination (Fig. S2D). MKN7 cells were co-cultured with macrophages stimulated by GCIY-EVs, and their migratory capacity was analyzed. These MKN7 cells were able to migrate more than MKN7 cells co-cultured with either macrophages without EV-treatment or those treated by MeT-5A cell-derived EVs (Fig. 3f and g). Moreover, macrophages treated by GCIY-EVs enhanced the migration ability of MKN7 cells more than macrophages treated by other GC-EVs (MKN7 or MKN45) (Fig. S3D).

Since it was reported that TAMs secrete IL-6 and promote malignancy of GC cells [25], whether macrophages increase secretion of IL-6 by stimulation of GC-EVs was then checked. In this assay, only M0-GM macrophages were used, because mature macrophages polarized from M0-M macrophages were unstable without cytokines or EVs. M0-GM macrophages were treated by each EV for 4 days to prepare mature macrophages. Analysis of the IL-6 level of the supernatant revealed that the macrophages treated by GC-EVs secreted more IL-6 than other macrophages (Fig. 3h).

Based on the results that GC-EVs were associated with skewing macrophages, and the resultant macrophages further secreted IL-6 and affected the phenotypes of cancer cells, GC-EVs must be playing a role in aggravating gastric cancer malignancy.

### EVs from a highly metastatic gastric cancer cell line carry STAT3, which was transferred into macrophages to change their phenotype

**Fig. 4:**
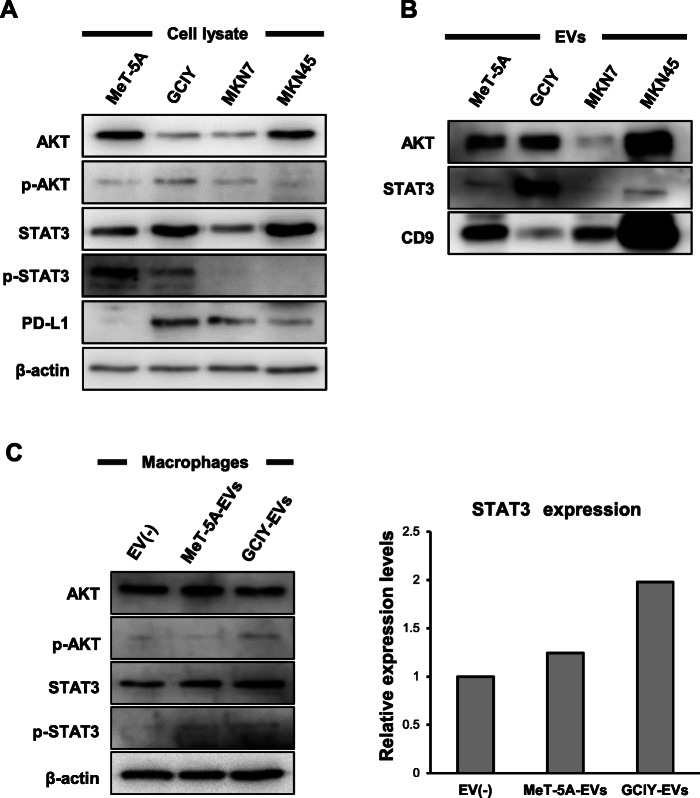
EVs from a high metastatic gastric cancer cell line carry STAT3 protein. **a** Western blotting for each cell lysate. 20 μg / lane. **b** Western blotting for EVs derived from each cell line. 2.5 μg / lane. **c** Western blotting for M0-GM macrophages treated by each EV. 30 μg / lane. Full-length blots / gels are presented in Fig. S8, S9 and S10

STAT3 or Akt expression levels in GC cell lines and EVs were examined. GCIY and MKN45, metastatic cell lines, expressed high levels of STAT3. GCIY-EVs contained more STAT3 than EVs from other GC cell lines. On the other hand, MKN7, a non-metastatic cell line, contained low levels of STAT3 and Akt (Fig. 4a and b). After treatment by GCIY-EVs (20 μg/ml), GM-M0 macrophages increased their STAT3 levels, although phosphorylated STAT3 levels were not elevated (Fig. 4c). STAT3 contained in GC-EVs might have a role in polarizing macrophages.

### EVs from malignant ascitic fluid affect macrophages, causing them to polarize into M2 phenotype

**Fig. 5:**
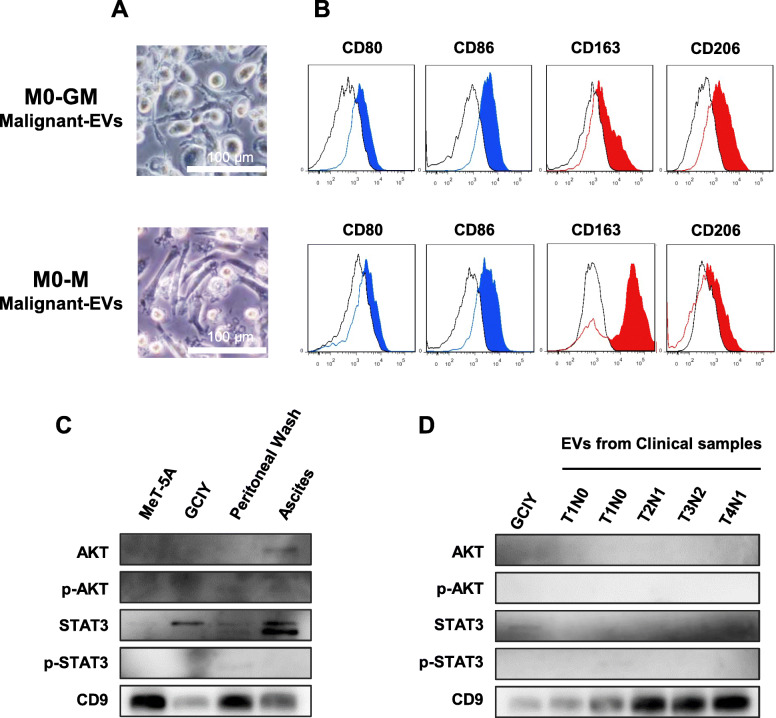
Macrophages were treated with EVs from clinical samples. **a** Morphology and **b** surface markers of macrophages stimulated by EVs contained in malignant ascitic fluid. Upper figures indicate macrophages differentiated from GM-M0, and lower figures indicate macrophages from M-M0. Scale Bar: 100 μm. **c** Western blotting for EVs from peritoneal washings of a GC patient with peritoneal nodules and malignant ascites. EVs from Met-5A and GCIY cells were applied for 1.5 μg, and EVs from clinical samples were applied for 1 μl of EV suspension. Full-length blots / gels are presented in Fig. S11. **d** Western blotting for EVs from peritoneal washings of a GC patient without peritoneal dissemination. EVs from cell lines were applied for 1.0 μg, and EVs from clinical samples were applied for 1.5 μg. Full-length blots / gels are presented in Fig. S12

Finally, whether EVs purified from clinical malignant ascitic fluid from GC patients with peritoneal dissemination (malignant EVs) have an effect on macrophages was examined. After GM-M0 or M-M0 macrophages were treated by malignant EVs for 4 days, the macrophages changed their morphology into M2-like ones (Fig. 5a), and the expression of CD163 was increased (Fig. 5b). Moreover, whether EVs purified from peritoneal washings or malignant ascitic fluid of GC patients contained STAT3 or Akt was also evaluated. STAT3 was detected in EVs from peritoneal washings of GC patients with disseminated nodules, as well as EVs from malignant ascitic fluid (Fig. 5c), while neither STAT3 nor Akt could be detected in EVs from patients without dissemination (Fig. 5d). These results suggested that STAT3 packed in EVs might be one of the mediators associated with skewing macrophage phenotypes.

## Discussion

In the present study, CD163+ M2 macrophages were predominant in the peritoneal cavity of GC patients. Since the underlying mechanism of preferential differentiation of macrophages remains to be clarified, whether EVs were secreted from GC cells and played a role in affecting monocytes or macrophages to polarize them was examined. EVs were isolated from the culture medium of GC cells, as well as clinical malignant ascitic fluid. Treatment with these EVs affected macrophages to make them more sustainable and skewed into a CD163+ type. Interestingly, the effect was observed regardless of whether they were on the process from monocyte to M1 or M2 macrophages. In addition, STAT3 protein was found to be carried in the EVs, indicating that STAT3 in the EVs might be a mediator between GC cells and monocytes/macrophages (Fig. S4).

Growing evidence suggests that the association between cancer-derived EVs and the tumor microenvironment must play a crucial role in the progression of various cancers. In the present study, monocytes treated by GC-EVs survived more, and most of them expressed CD163 on their surface, which might be one of the reasons why CD163+ cells were predominant in malignant ascitic fluid. Moreover, these monocytes were expressing PD-L1.

PD-L1 on macrophages might bind to PD-1 on T cells and suppress T cell-mediated cancer immunity, as previously shown in glioblastoma [36]. In addition, monocytes polarized to the M2 macrophage phenotype upregulate PD-L1 and elaborate cytokines, such as MCP-3 and CXCL1, which further enhance immune infiltration, the induction of angiogenesis, and the recruitment of myeloid cells into the tumor microenvironment [37, 38]. Therefore, these situations might be caused by GC-derived EVs and contribute to maintaining the immune-suppressive microenvironment in the peritoneal cavity, thereby supporting further peritoneal spread of GC.

In addition, macrophages co-cultured with GCIY-EVs increased the migration ability of cancer cells. TAMs are known to promote the malignant potential of cancer cells, as previously reported [22, 25], consistent with the present results, and it is suggested that cancer-derived EVs are possible mediators between tumor-associated macrophages and cancer progression.

It had been reported that various factors secreted from cancer cells including cytokines and chemokines, as well as microRNAs in EVs, were involved in differentiation into M2 macrophages or TAM to promote cancer malignancy [22, 39–43]. To polarize macrophages into the M2 type, the STAT3 pathway and the Akt-mTOR pathway are known pathways [44, 45]. Although our data showed that STAT3 was contained in GC-derived EVs, a recent report demonstrated that STAT3 protein packaged in EVs was transferred from glioblastoma stem cells into macrophages to polarize them into the M2 phenotype [36]. The results suggested that EVs from cancer cells might induce M2 macrophages through the STAT3 pathway. Yu and coworkers also reported that CD11b + myeloid precursor cells derived from bone marrow were stimulated by exosomes from murine mammary tumor cells and upregulated STAT3 [46]. The persistent activation of STAT3 mediates tumor-promoting inflammatory pathways, including nuclear factor-κB (NF-κB) and interleukin-6 (IL-6)–GP130–Janus kinase (JAK) pathways [47]. STAT3 is commonly activated in tumor-infiltrating macrophages [48, 49], and STAT3 can bind to the promoter of the CD274 gene required for PD-L1 gene expression in T-cell lymphoma [38, 50]. Therefore, STAT3 activation might also contribute to inducing PD-L1 in macrophages [51]. On Western blotting, STAT3 protein could be detected in EVs purified from not only malignant ascitic fluid, but also peritoneal washings of cytology-positive GC patients, while STAT3 could not be found in EVs from cytology-negative GC patients. These data suggest that cancer cells that infiltrated into the peritoneal cavity might secrete EVs containing STAT3 and promote polarization into M2 macrophages.

However, it should be recognized that STAT3 would not the only pathway that skews macrophages to M2 type, given that EVs carry multiple proteins and nucleic acids that affect cell function [52]. According to the review by Chatterjee et al., several microRNAs packaged in EVs from cancer cells have recently been reported to regulate this process [53]. For instance, EVs containing miR-222-3p or miR-940 derived from ovarian cancer cells induce the M2-like phenotype. Colon cancer cells secrete EVs enriched in miR-1246 or miR-203, thereby promoting M2 polarization of macrophages. Several other EVs-loaded microRNAs, such as miR-let7a, miR-21, miR-125b-5p, miR-21-2p, miR181d-5p, and miR-503 have also been reported to induce M2-like phenotype in macrophages. Thus, emerging studies have shown that tumor cells actually secrete EVs which skew macrophages to protumor phenotypes, which is consistent with our results. On the other hand, it is also suggested that there are multiple pathways in the process.

It has recently been reported that cancer cells polarize macrophages into the M2 phenotype in co-culture, and such phenotype changes make macrophages secrete IL-6, which promotes peritoneal dissemination [25]. In the present study, M2-macrophages polarized by EVs also secreted IL-6 to promote malignancy of cancer cells. Collectively, the EVs from cancer cells might play crucial roles in boosting the malignant potentials of GC and in the promotion of peritoneal dissemination.

​In this study, we used CD9, CD 63, and CD 81 as markers of EVs, but the expression intensities of these markers differed in each sample. Proteins enriched in EV are often used as markers include tetraspanins (CD9, CD63, CD81 and CD82), 14–3-3 proteins, major histocompatibility complex molecules and cytosolic proteins such as heat shock proteins [15]. Although CD9 and CD81 belong to the most frequently identified EV proteins [54], abundance of CD63 was reported to be variable depending on the cell types [55]. Recently A comprehensive proteomic analysis of EVs from large number of human samples was reported [52]. Among conventional markers, heat shock cognate 71 kDa protein (HSPA8), heat shock protein HSP 90-beta (HSP90AB1), CD9, and programmed cell death 6-interacting protein (ALIX) appeared to be the most prominent markers found in human-derived EVs isolated from cells, tissues, and body fluids. It is necessary to recognize that the panel of human EV markers are drastically expanding by the progress of the EV research.

This study has potential limitations that need to be addressed. First, macrophage phenotype was classified by the M1-M2 paradigm, and it was considered that TAMs are generally closer to M2-polarized macrophages. However, macrophages actually show a spectrum of activation states, rather than such dichotomous phenotypes [56]. Although this study was based on the M1 and M2 classification scheme, it was chosen to help simplify the understanding of the association between EVs and macrophages. It should be recognized that macrophages are plastic and versatile, and the concept of M1-M2 extremes may not capture their whole spectrum [56–59]. Second, based on the results of the present study, it was assumed that the macrophages treated with EVs produced IL-6. The previous studies demonstrated that the main producers of IL-6 are the myeloid cells [60, 61]. However, autocrine IL-6 in epithelial cancer cells has also been documented [62, 63]. Thus, cells other than peritoneal macrophages may also secrete IL-6 under certain conditions. Third, common problems in experiments with EVs must be taken into account. The amount of protein contained in EVs from some GC patients was too low to be subjected to the analysis, which did not allow, for example, checking the STAT3 level in all clinical samples. The purification and concentration process of EVs needs to be improved for further investigations. Finally, although monocytes isolated from one volunteer were used in this study, analysis of monocytes from many volunteers would be ideal for obtaining more meaningful data.

## Conclusions

EVs derived from GC cells could induce polarization via the STAT3 pathway into M2-macrophages, which secrete IL-6 to promote malignancy of cancer cells. EVs derived from GC play a role by affecting macrophage phenotypes, and this may be a part of the underlying mechanism that forms the intraperitoneal cancer microenvironment.

## Supplementary Information

**Additional file 1: Figure S1.** (A) Transmission electron microscope image of EVs. Scale bar: 500 nm. (B) Vesicle size was measured by dynamic light scattering analyses.

**Additional file 2: Figure S2.** Extracellular vesicles derived from MKN7 (upper figure) and MKN45 (lower figure). (A) Transmission electron microscope image of EVs. Scale bar: 500 nm. (B) Vesicle size was measured by dynamic light scattering analyses. (C) Representative Western blot of original cell lysates (5 μg / lane) and EVs (1 μg / lane) to confirm the presence of EVs by exosome markers (CD9, CD63, CD81). Full-length blots / gels are presented in Fig. S13 and S14. (D) Mouse-dissemination model for each GC cell line. (E) The fluorescence microscope images of GCIY-EVs taken up into CD14+ cells (monocytes) at 48 h after co-incubation. Scale bar: 100 nm. (F) The uptake of GCIY-EVs into CD14-negative cells (mainly lymphocytes) was analyzed by flow cytometry.

**Additional file 3: Figure S3.** (A) Protocol of M1 or M2 macrophage polarization. (B) Morphology of M1 (upper) or M2 (lower) macrophages. Scale bar: 100 nm. (C) Surface marker of M1 (upper) or M2 (lower) macrophages analyzed by flow cytometry. (D) Migration assay of MKN7 cells co-cultured with macrophages stimulated by each cancer cell EVs.

**Additional file 4: Figure S4.** A schematic model of GC-derived EVs that promote macrophage differentiation and thereby promote GC migration. GC cell-derived EVs containing STAT3 mediate M2 polarization of macrophages and promote migration of GC cells through IL − 6 secretion.

**Additional file 5: Figure S5.** The uncropped full-length gels and blots for Fig. 1e. **Figure S6.** The uncropped full-length gels and blots for Fig. 2c upper. **Figure S7.** The uncropped full-length gels and blots for Fig. 2c lower. **Figure S8.** The uncropped full-length gels and blots for Fig. 4a. **Figure S9.** The uncropped full-length gels and blots for Fig. 4b. **Figure S10.** The uncropped full-length gels and blots for Fig. 4c. **Figure S11.** The uncropped full-length gels and blots for Fig. 5c. **Figure S12.** The uncropped full-length gels and blots for Fig. 5d. **Figure S13.** The uncropped full-length gels and blots for Fig. S2C upper. **Figure S14.** The uncropped full-length gels and blots for Fig. S2C lower.

**Additional file 6: Supplementary Table.** Additional information on cell lines used in the experiments.

## Data Availability

The datasets used and/or analyzed during the current study are available from the corresponding author on reasonable request.

## Abbreviations

TAMs: Tumor-associated macrophages
EVs: extracellular vesicles
GC: Gastric cancer
TME: tumor microenvironment
miRNAs: microRNAs
CM: conditioned medium
TEM: Transmission electron microscopy
PBMCs: peripheral blood mononuclear cells
DAPI: 4′, 6-diamidino-2-phenylindole
mAb: monoclonal antibody
STAT3: signal transducer and activator of transcription 3
pAkt: phosphorylated Akt
PET: polyethylene terephthalate
ELISA: an enzyme-linked immunosorbent assay
GCIY-EVs: EVs purified from GCIY culture medium
GC-EVs: GC cell-derived EVs
NF-κB: nuclear factor-κB
IL-6: interleukin-6
JAK: Janus kinase
