# Supplementary figures and images for: Extracellular vesicles shed from gastric cancer mediate protumor macrophage differentiation
Source: BMC Cancer. 2021 Jan 28;21:102. doi: 10.1186/s12885-021-07816-6 (PMC7845052; doi:10.1186/s12885-021-07816-6)

Figure S1

**A**

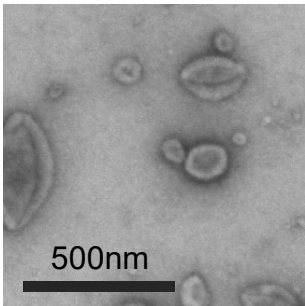

**B**

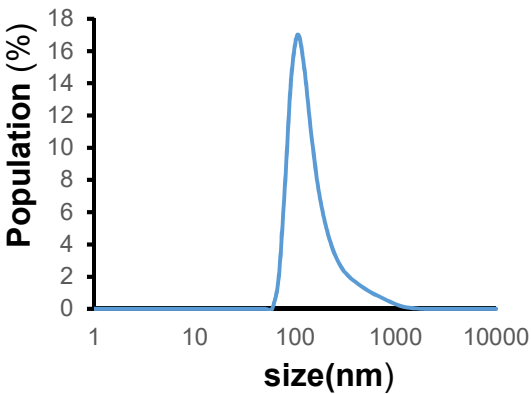

Supplement: Supplementary file 1 — Additional file 1: Figure S1. (A) Transmission electron microscope image of EVs. Scale bar: 500 nm. (B) Vesicle size was measured by dynamic light scattering analyses. [file 12885_2021_7816_MOESM1_ESM.pdf]

Figure S2

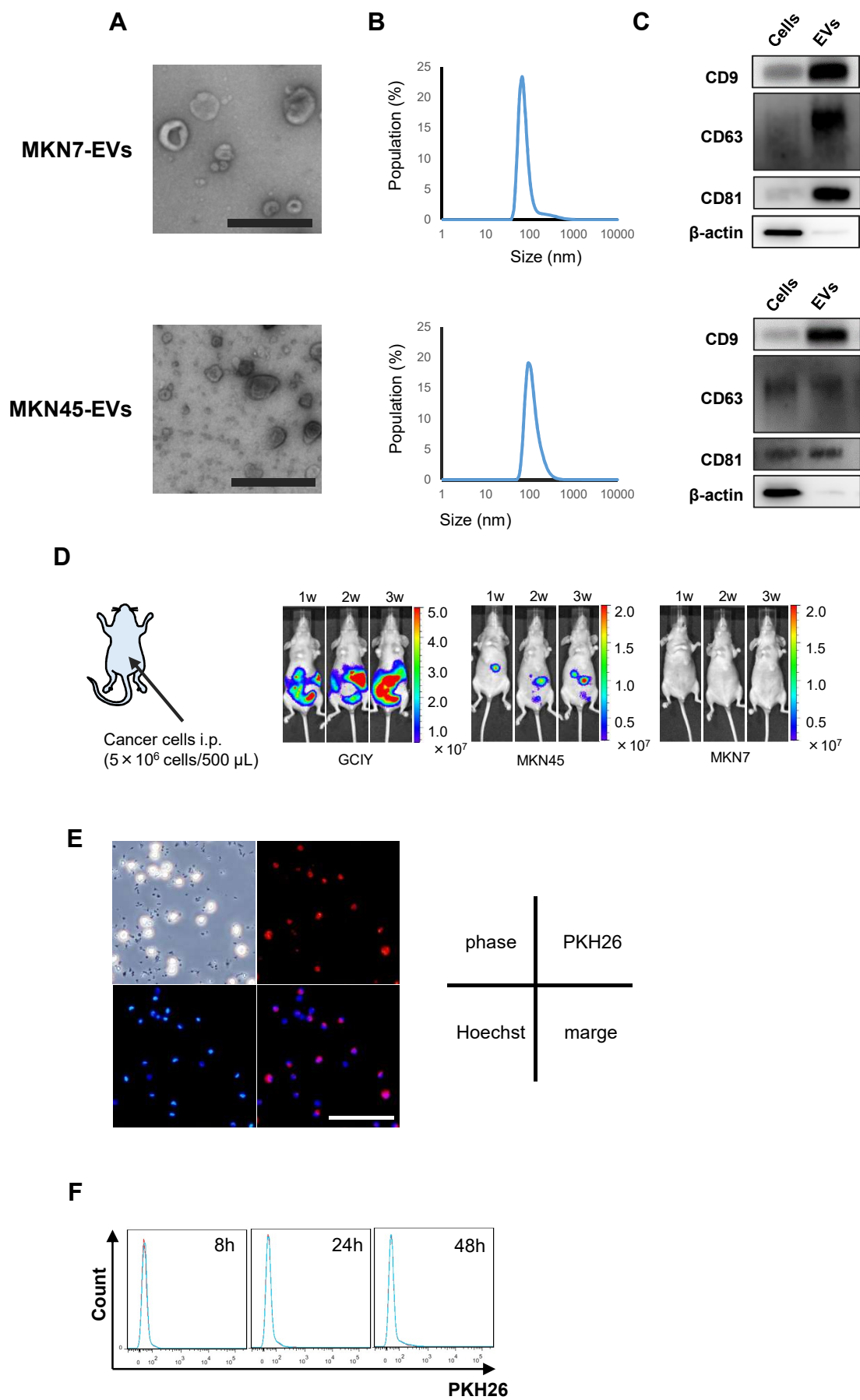

Supplement: Supplementary file 2 — Additional file 2: Figure S2. Extracellular vesicles derived from MKN7 (upper figure) and MKN45 (lower figure). (A) Transmission electron microscope image of EVs. Scale bar: 500 nm. (B) Vesicle size was measured by dynamic light scattering analyses. (C) Representative Western blot of original cell lysates (5 μg / lane) and EVs (1 μg / lane) to confirm the presence of EVs by exosome markers (CD9, CD63, CD81). Full-length blots / gels are presented in Fig. S13 and S14. (D) Mouse-dissemination model for each GC cell line. (E) The fluorescence microscope images of GCIY-EVs taken up into CD14+ cells (monocytes) at 48 h after co-incubation. Scale bar: 100 nm. (F) The uptake of GCIY-EVs into CD14-negative cells (mainly lymphocytes) was analyzed by flow cytometry. [file 12885_2021_7816_MOESM2_ESM.pdf]

Figure S3

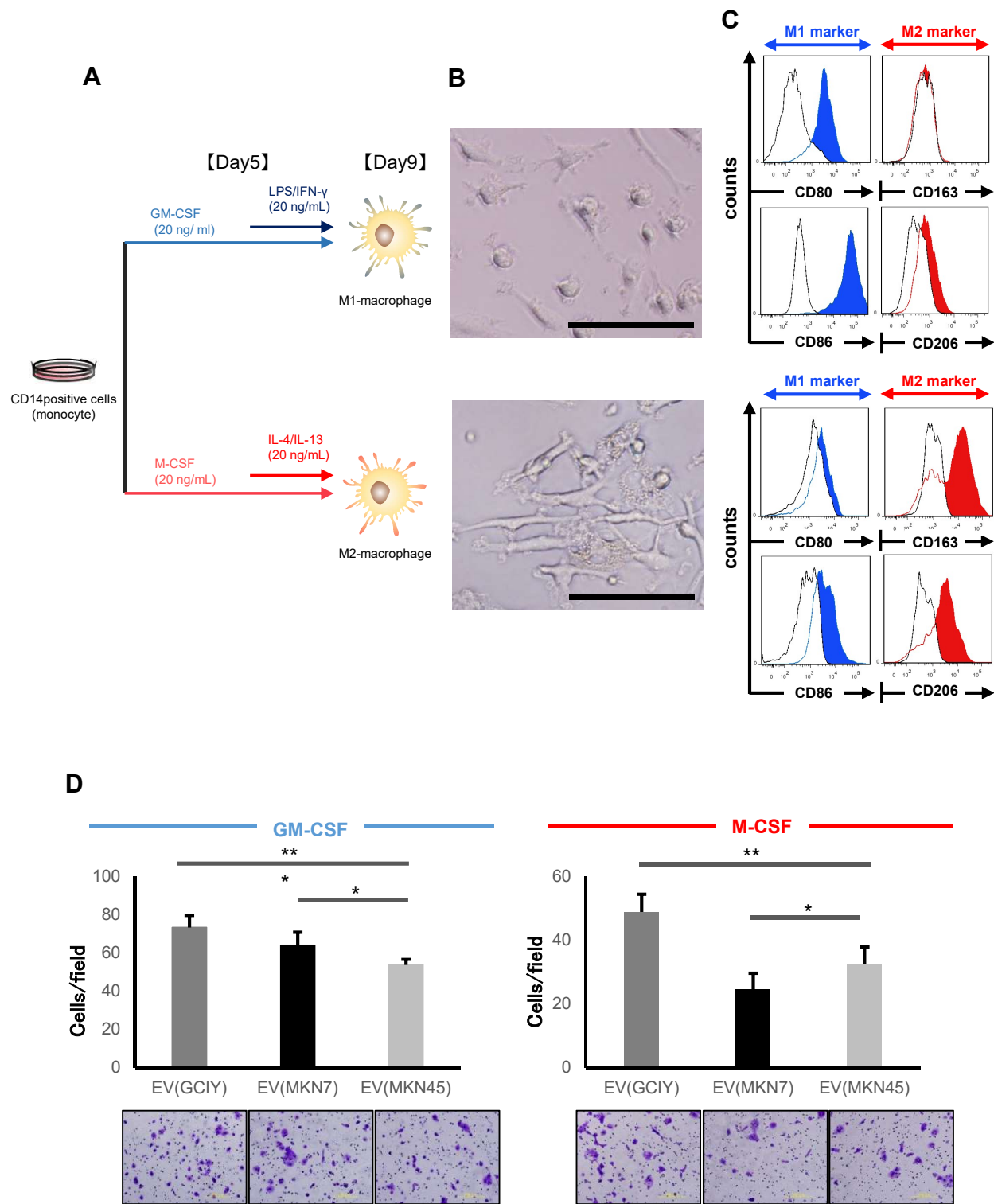

Supplement: Supplementary file 3 — Additional file 3: Figure S3. (A) Protocol of M1 or M2 macrophage polarization. (B) Morphology of M1 (upper) or M2 (lower) macrophages. Scale bar: 100 nm. (C) Surface marker of M1 (upper) or M2 (lower) macrophages analyzed by flow cytometry. (D) Migration assay of MKN7 cells co-cultured with macrophages stimulated by each cancer cell EVs. [file 12885_2021_7816_MOESM3_ESM.pdf]

Figure S4

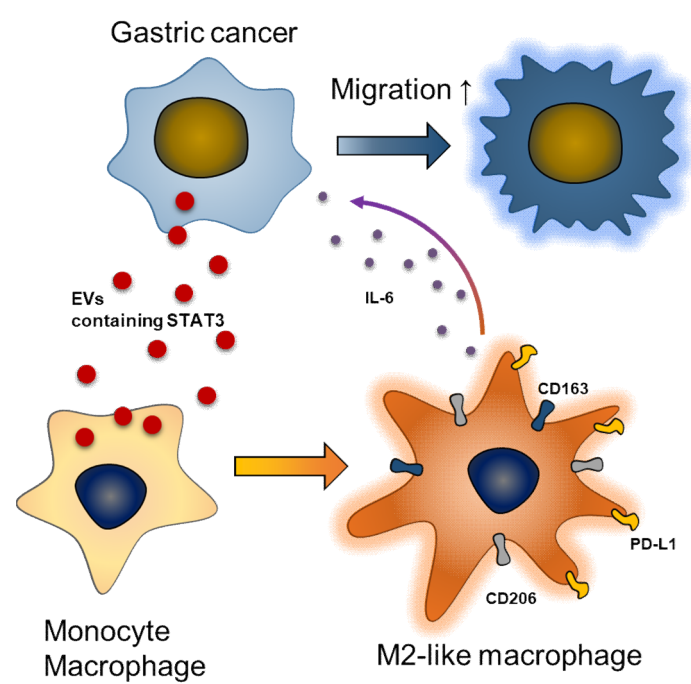

Supplement: Supplementary file 4 — Additional file 4: Figure S4. A schematic model of GC-derived EVs that promote macrophage differentiation and thereby promote GC migration. GC cell-derived EVs containing STAT3 mediate M2 polarization of macrophages and promote migration of GC cells through IL − 6 secretion. [file 12885_2021_7816_MOESM4_ESM.pdf]
