# Supplementary material for: Extracellular vesicles shed from gastric cancer mediate protumor macrophage differentiation
Source: BMC Cancer. 2021 Jan 28;21:102. doi: 10.1186/s12885-021-07816-6 (PMC7845052; doi:10.1186/s12885-021-07816-6)

Figure S5

Figure 1E

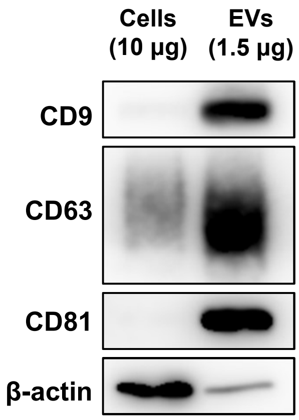

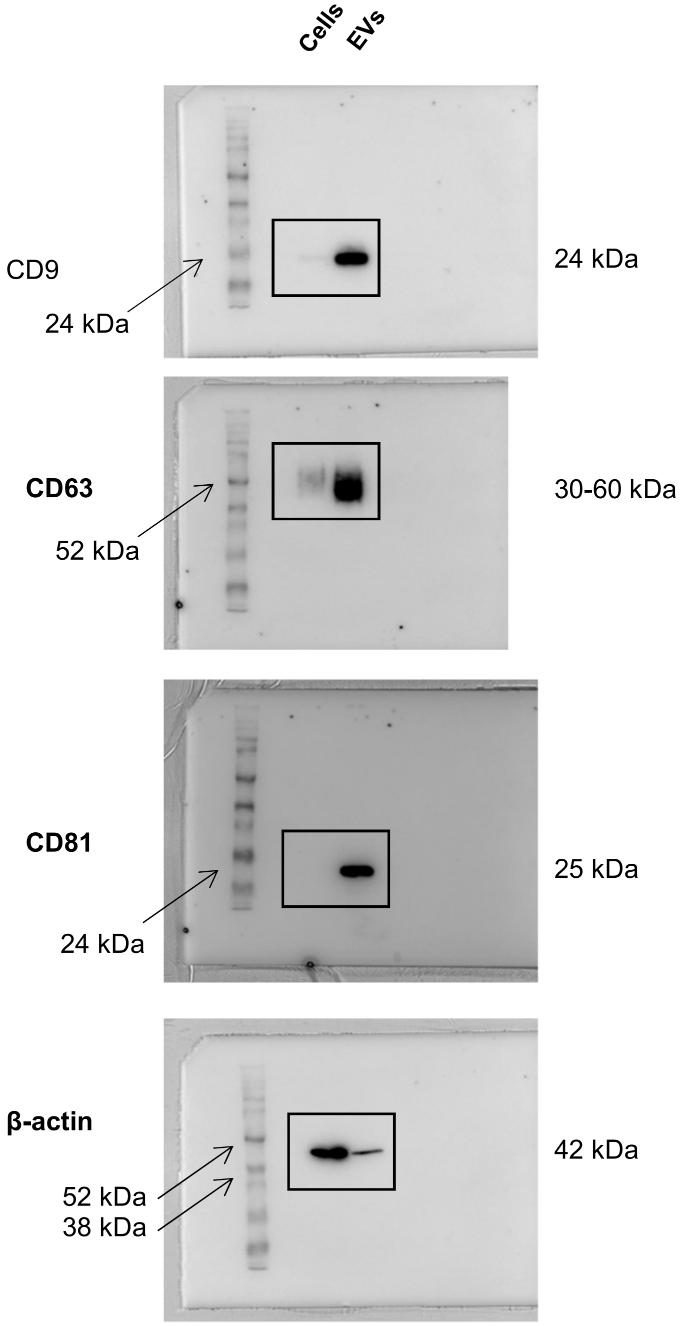

Figure S6

Figure 2C upper

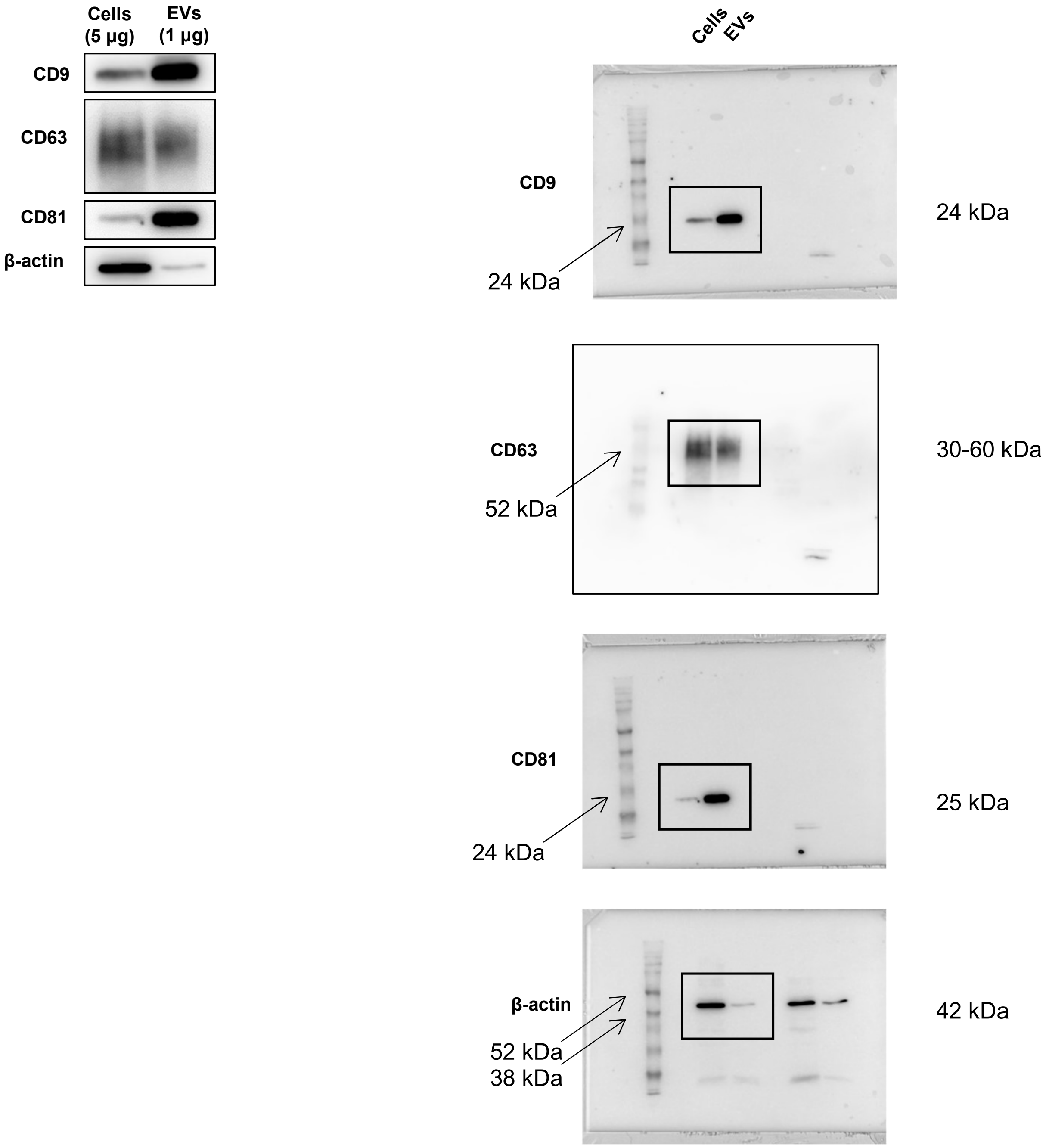

Figure S7

Figure 2C lower

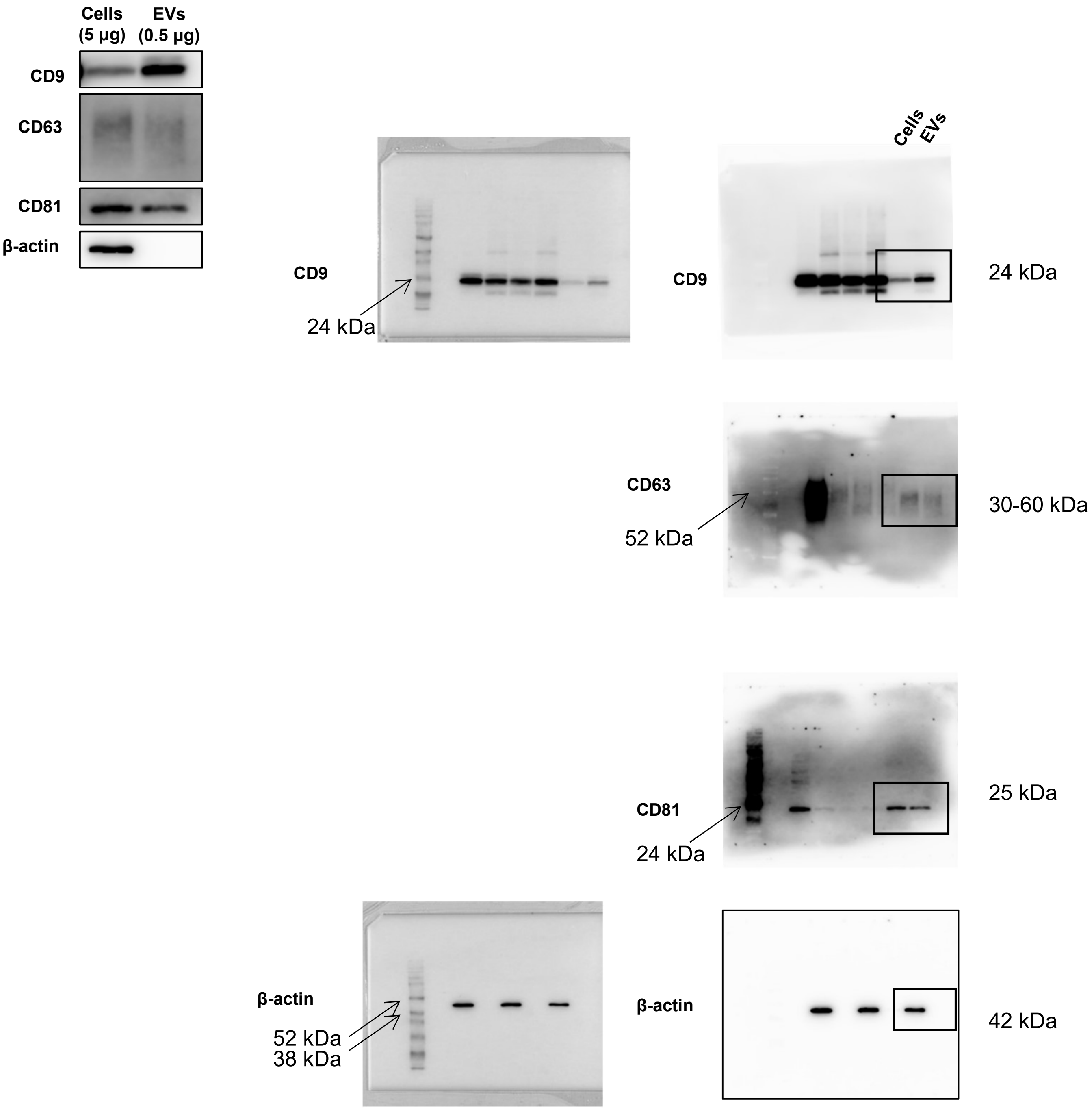

Figure S8

Figure 4A

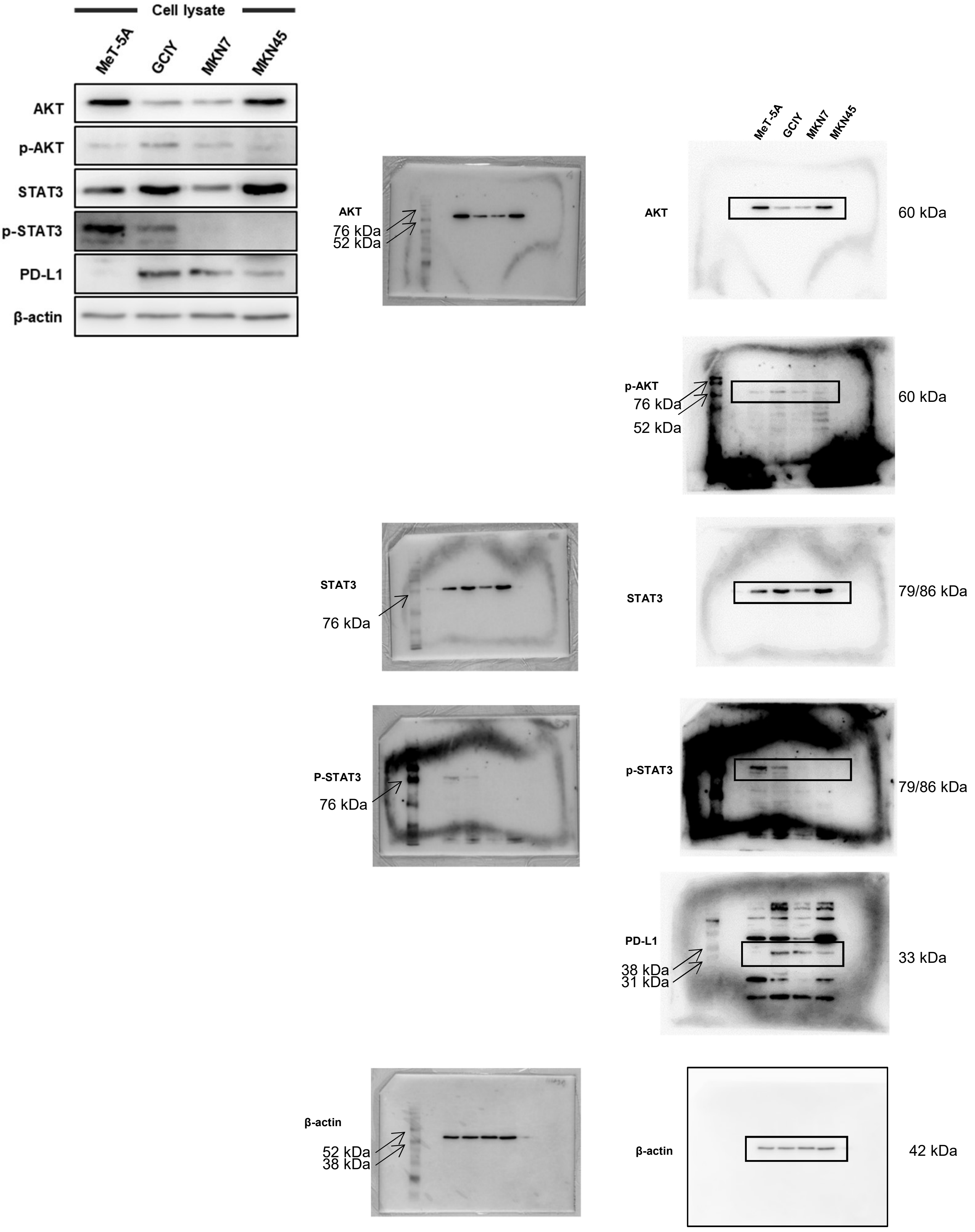

Figure S9

Figure 4B

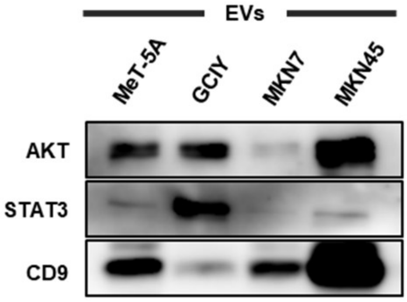

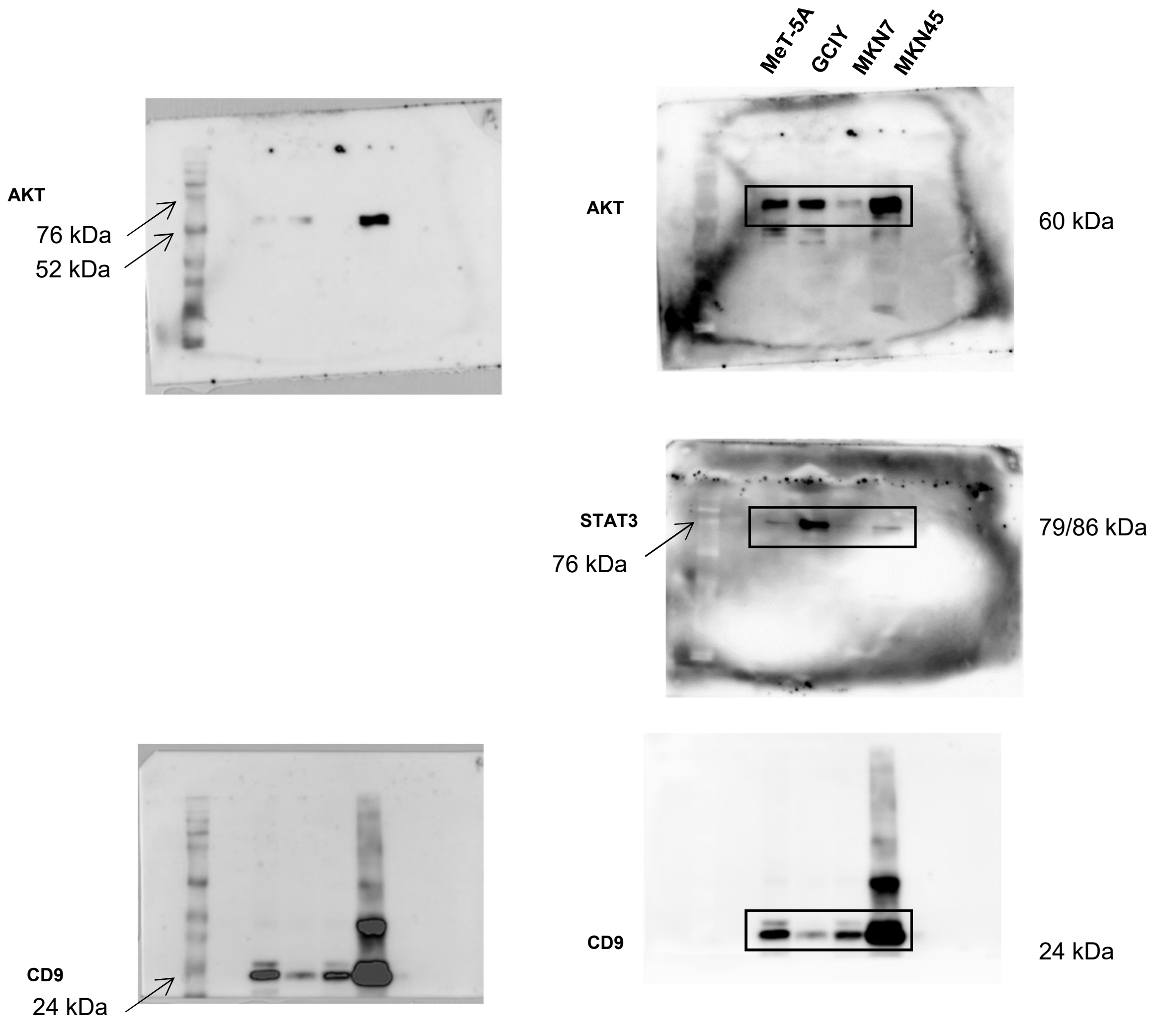

Figure S10

Figure 4C

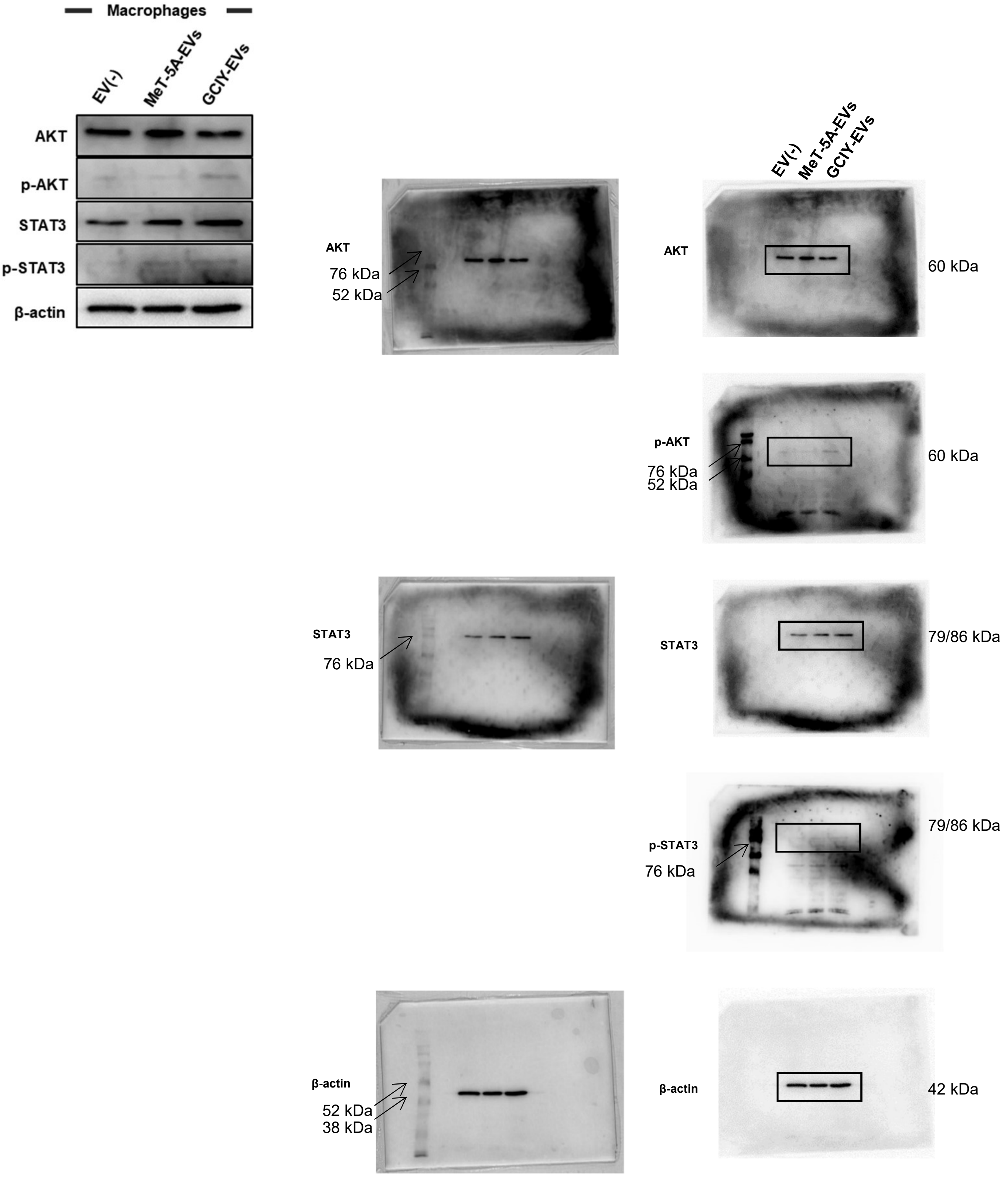

Figure S11

Figure 5C

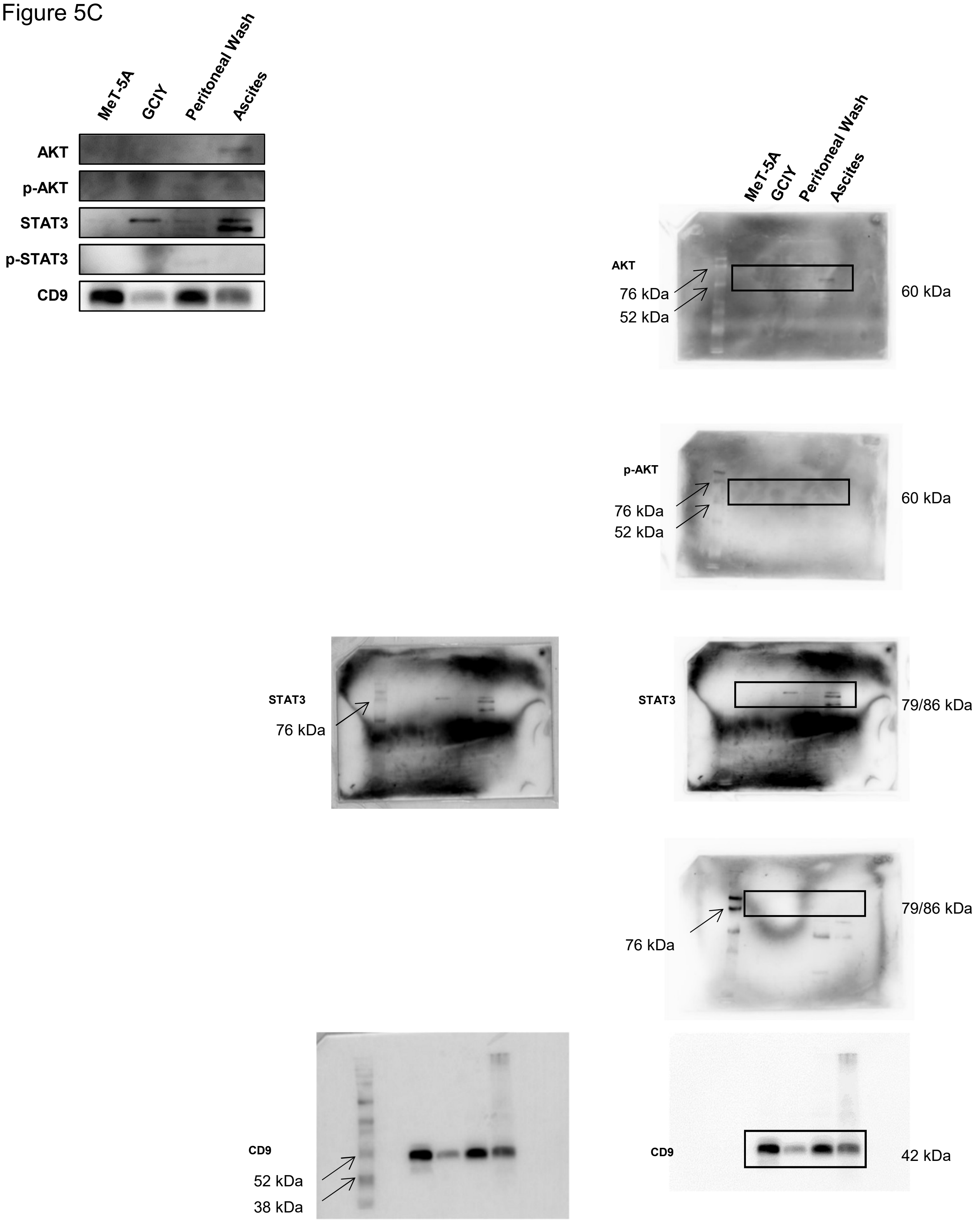

Figure S12

Figure 5D

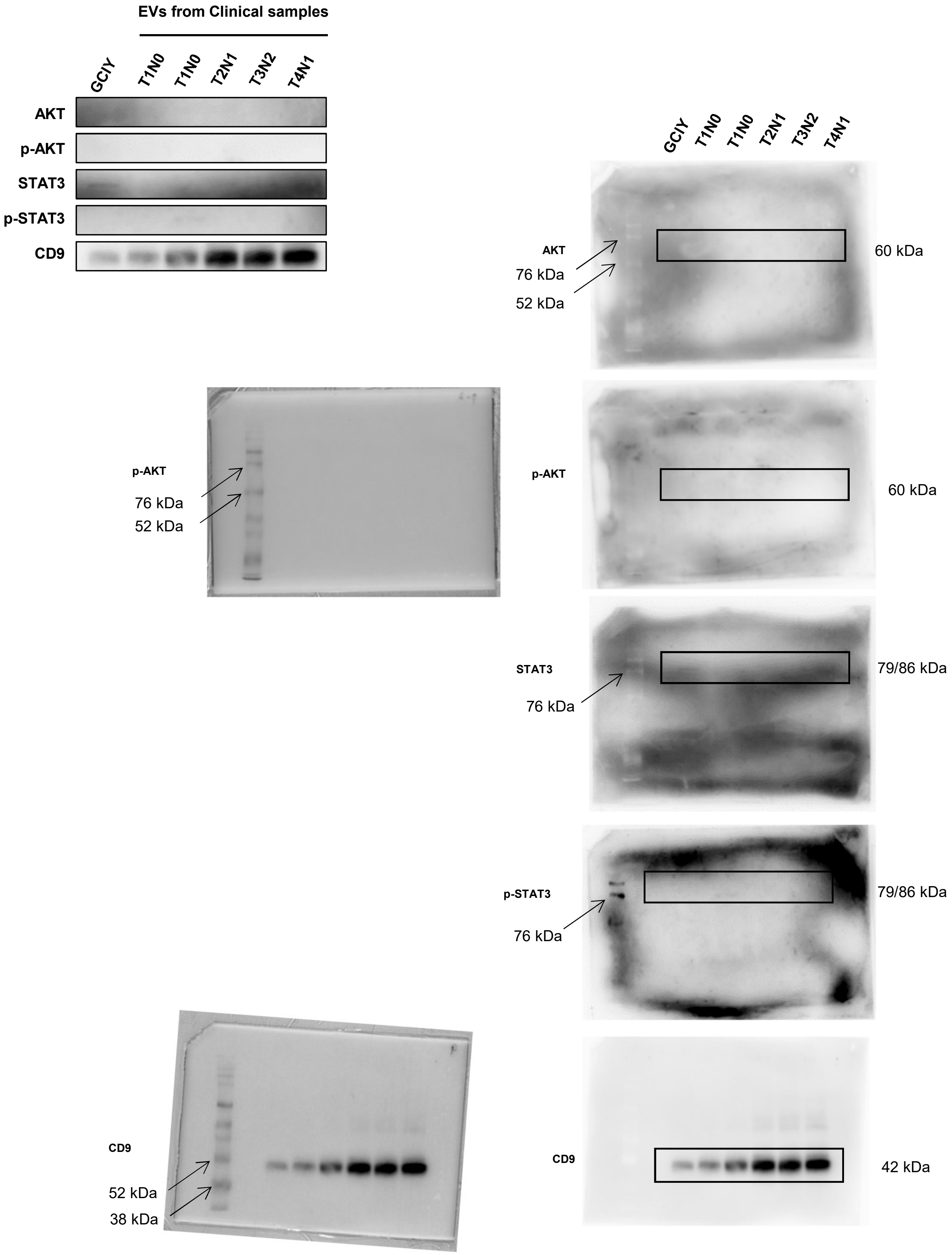

Figure S13

Figure S2C upper

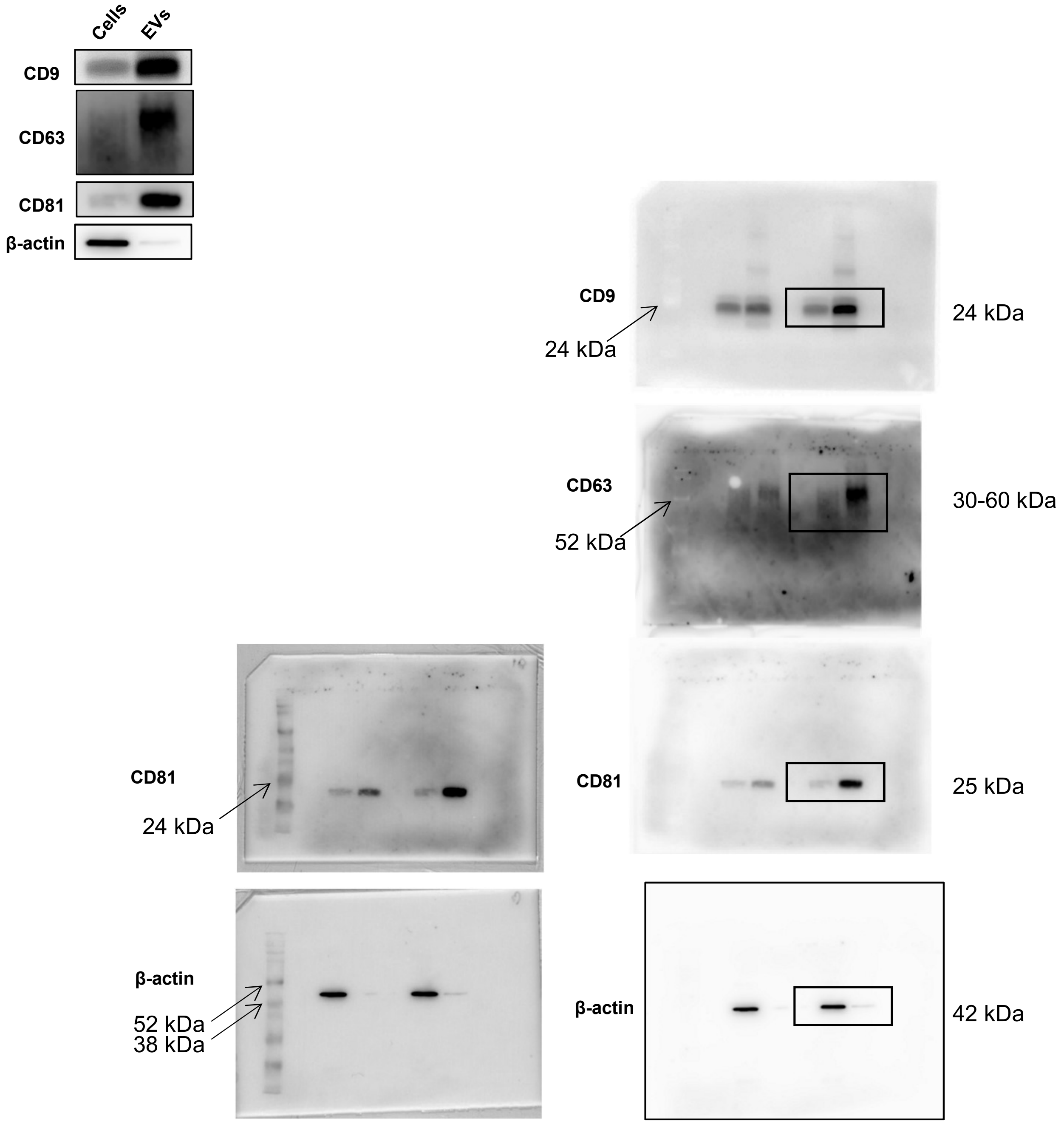

Figure S14

Figure S2C lower

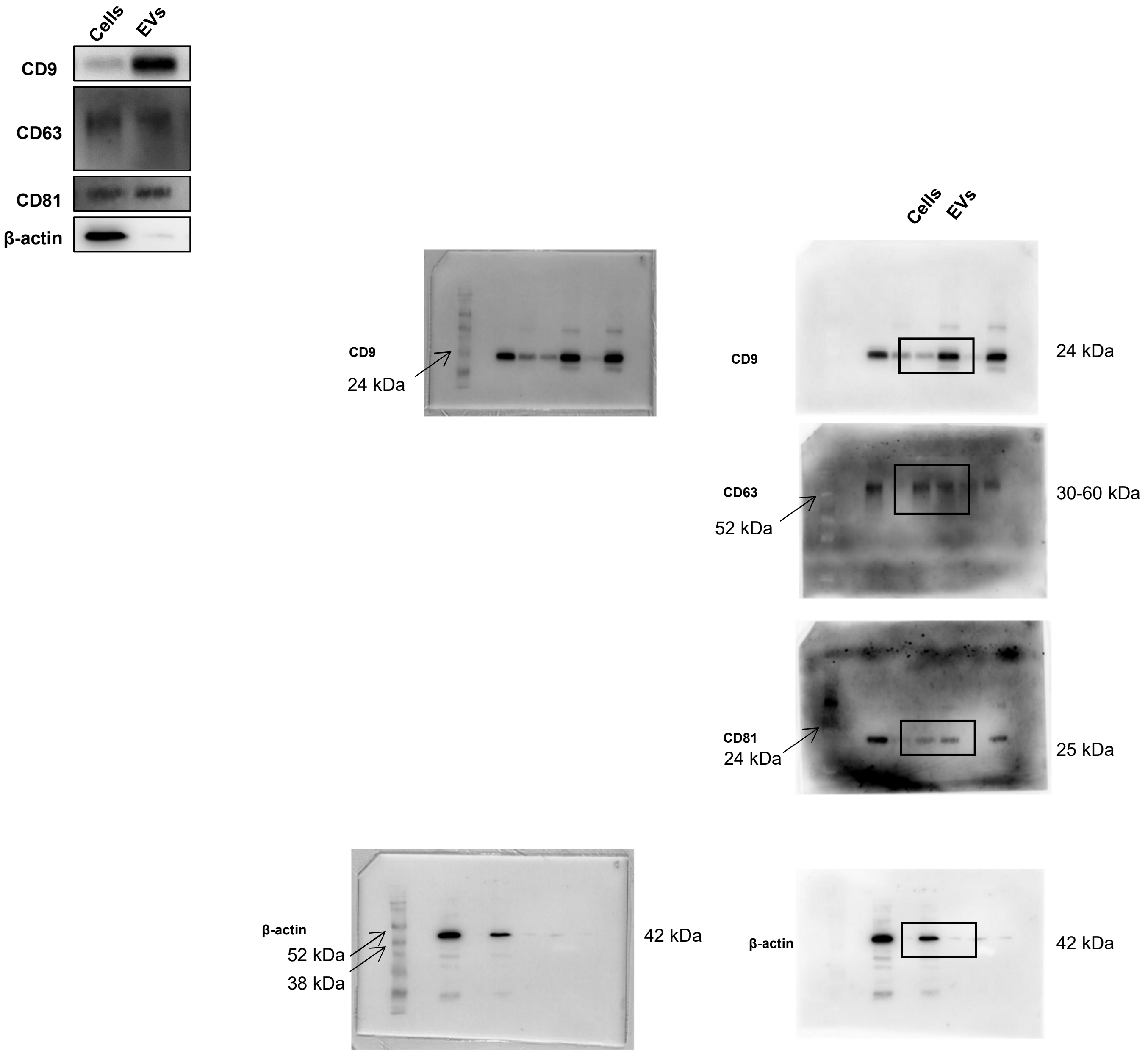

Supplement: Supplementary file 5 — Additional file 5: Figure S5. The uncropped full-length gels and blots for Fig. 1e. Figure S6. The uncropped full-length gels and blots for Fig. 2c upper. Figure S7. The uncropped full-length gels and blots for Fig. 2c lower. Figure S8. The uncropped full-length gels and blots for Fig. 4a. Figure S9. The uncropped full-length gels and blots for Fig. 4b. Figure S10. The uncropped full-length gels and blots for Fig. 4c. Figure S11. The uncropped full-length gels and blots for Fig. 5c. Figure S12. The uncropped full-length gels and blots for Fig. 5d. Figure S13. The uncropped full-length gels and blots for Fig. S2C upper. Figure S14. The uncropped full-length gels and blots for Fig. S2C lower. [file 12885_2021_7816_MOESM5_ESM.pdf]
