# Supplementary material for: Extracellular vesicles shed from gastric cancer mediate protumor macrophage differentiation
Source: BMC Cancer. 2021 Jan 28;21:102. doi: 10.1186/s12885-021-07816-6 (PMC7845052; doi:10.1186/s12885-021-07816-6)
Supplement: Supplementary file 6 — Additional file 6: Supplementary Table. Additional information on cell lines used in the experiments. [file 12885_2021_7816_MOESM6_ESM.pdf]

Cell line	The source	Catalogue No.	Lot No.	Date received	Test for mycoplasma
GCIY	RIKEN Cell Bank	RBRC-RCB0555	005	22 Jul, 2014	negative
MKN7	JCRB	JCRB1025	02102011	10 Jun, 2014	negative
MNK45	JCRB	JCRB0254	05242012	31 Jan, 2017	negative
MeT-5A	ATCC	CRL-9444	63990138	5 Oct, 2017	negative
